# The Policy Effect of the General Data Protection Regulation (GDPR) on the Digital Public Health Sector in the European Union: An Empirical Investigation

**DOI:** 10.3390/ijerph16061070

**Published:** 2019-03-25

**Authors:** Bocong Yuan, Jiannan Li

**Affiliations:** Faculty of Economics and Management, Sun Yat-sen University, West Xingang Rd. 135, Guangzhou 510275, China

**Keywords:** digital health, General Data Protection Regulation (GDPR), financial performance, information and communication technologies (ICT), hospitals, healthcare organizations, European Union

## Abstract

The rapid development of digital health poses a critical challenge to the personal health data protection of patients. The European Union General Data Protection Regulation (EU GDPR) works in this context; it was passed in April 2016 and came into force in May 2018 across the European Union. This study is the first attempt to test the effectiveness of this legal reform for personal health data protection. Using the difference-in-difference (DID) approach, this study empirically examines the policy influence of the GDPR on the financial performance of hospitals across the European Union. Results show that hospitals with the digital health service suffered from financial distress after the GDPR was published in 2016. This reveals that during the transition period (2016–2018), hospitals across the European Union indeed made costly adjustments to meet the requirements of personal health data protection introduced by this new regulation, and thus inevitably suffered a policy shock to their financial performance in the short term. The implementation of GDPR may have achieved preliminary success.

## 1. Introduction

### 1.1. Contextualization

Public health increasingly appears to be a science of information [1]. Having entered the digital health age, a growing number of hospitals and healthcare institutions are embracing information and communication technologies (ICTs) to support and advance their healthcare practices. For patients, it has become common practice to go online to search health information [2]. In this context, a wide range of e-health tools and services have emerged. Electronic health records are built to enable the communication of patient data between different healthcare professionals. Telemedicine improves access to medical services by using ICTs to provide physical and psychological treatment at a distance. Internet health platforms are created with a series of powerful functions, including providing healthcare information to practitioners, researchers and patients, collecting aggregate and patient-level health data, and printing e-prescriptions to patients.

Although digital health has so many meaningful applications, its high dependence on sensitive information with respect to patients’ personal health can inevitably trigger data security problems. Vast amounts of personal health data have been collected, often without of the awareness of patients. Patients cannot control their data, and do not have sufficient levels of awareness to give free and informed consent [3]. The development of complex algorithm systems has further aggravated this problem, since it enables invasive inferences about health conditions of patients, bringing serious risks for personal health data protection [4]. 

These potential risks of digital health have received the attention of legislators. In legislative efforts for protecting personal privacy, Europe has been at the forefront [5]. The most notable example is the European Union General Data Protection Regulation (GDPR), the most recent piece of legislation on data protection, which was passed in April 2016 and came into force in May 2018 across the European Union. The GDPR aims to overhaul previous data protection laws in Europe [3], and in doing so cope with the new challenges of data protection in the digital health era. It makes efforts to clarify personal health data protection in digital healthcare delivery [6] where boundaries and responsibilities are less clear than in traditional healthcare delivery [7]. It involves a fundamental change in control and ownership of health data—from physicians, scientists, hospitals, and healthcare institutions, to patients [8,9,10]. The patients must now give their explicit consent for use of their health data, and can withdraw it whenever needed. As such, in comparison with previous regulations on health data protection, the GDPR devotes much more efforts to meet the new and higher requirements raised by the digital health era, and may thus contribute to stronger health data protection across the European Union. 

### 1.2. The Importance of the Theme

Facing the challenges of health data protection in the digital health age, this new regulation aims to address them and advance the level of health data protection in the European Union. Yet, it is unclear whether this legal reform is effectively implemented by public health sectors across the European Union [11]. It is thus essential to test the effectiveness of the European Union GDPR for personal health data protection. Test results can help legislators understand the actual policy effect of this new regulation, and then help them take corresponding measures to promote its implementation by public health sectors across the European Union. 

### 1.3. The Proposal of the Research Question

On the one hand, the GDPR’s implementation by hospitals and healthcare institutions across the European Union can reflect the actual effectiveness of the GDPR for personal health data protection. It is thus necessary to examine the current levels of compliance with the GDPR by these healthcare organizations. Before becoming enforceable in May 2018, the GDPR was published in April 2016. This transition period from April 2016 to May 2018 was allowed for making preparations for this new legislation. Hospitals and healthcare institutions across the European Union needed to make adjustments for the GDPR in various ways, such as staff training on the GDPR, staff recruitment of some key GDPR roles, the purchase of equipment, and system installation for the new requirements of the GDPR. The costly adjustments may have led to financial distress, but the temporary financial distress reflects their efforts in preparation. Hence, by making comparison of financial performance before and after 2016, the effectiveness of this regulation can be accurately tested. 

On the other hand, the adjustment for this new legislation by these organizations actually indicates that their levels of personal health data protection before the GDPR came into effect had not spontaneously reached the new standards introduced by the GDPR. Such an existing gap, again, shows the effectiveness and necessity of the GDPR for personal health data protection. The GDPR is needed to remedy deficiencies in personal health data protection. Its articles should reflect the current limitations and further improvement directions of personal health data protection for hospitals and healthcare institutions across the European Union. Thus, the possible financial distress resulting from costly adjustments can be considered as measurable evidence to verify the existence of the gap in personal health data protection and then reflect the effectiveness of the GDPR. 

As such, to test the effectiveness of the European Union GDPR for personal health data protection, this study will empirically examine the effect of the GDPR on financial performance of hospitals and healthcare institutions across the European Union using the difference-in-difference (DID) approach. The digital health business is not viewed as being equally important for all hospitals across the European Union. Some hospitals regard the information, communication, and telecommunication business surrounding health as the largest primary business aside from the provision of common hospital services, while others do not. Based on the practice recommended by the difference-in-difference (DID) approach [12], the former can be viewed as the treatment group and the latter as the control group. This approach is used to compare the impacts of the GDPR shock on financial performance between these two groups. The statistically significant results of comparison can give an answer to our research question. 

### 1.4. Objectives

The present study is the first empirical research to test the effectiveness of the European Union GDPR for personal health data protection. The test results will reveal the aggregate level of the GDPR’s implementation by public health sectors across the European Union, and from other side reflect the existing level of personal health data protection before the introduction of the GDPR. The empirical findings can help legislators grasp the situation of current preparations for the GDPR, whereby they will make plans to further promote comprehensive implementation of the GDPR. 

### 1.5. The Structure of This Paper

This paper is structured as follows. In the following sections, this paper first describes the development of digital health, and analyzes the challenges for personal health data protection which the digital health is facing. Then this paper discusses the new requirements and standards introduced by the European Union GDPR in protecting personal health data. Combining these two sections, this paper then analyzes the potential impacts of GDPR shock on operations of public health sectors across the European Union. In the following section of method, this paper gives an empirical examination to reveal the actual effectiveness of the GDPR in protecting personal health data across the European Union. To clarify the difference-in-difference (DID) approach, this paper offers the relevant technique details. Meanwhile, this paper makes a series of robust tests to confirm the robustness of our findings. Finally, in the last section, this study concludes that the existing conditions of design and deployment of healthcare technologies have not fully met new standards introduced by the GDPR in relation to personal health data protection. The European Union GDPR is indeed represented a significant shock for the financial performance of public health sectors across the European Union. There is a need for urgent and further adjustments in design and deployment of healthcare technologies to ensure the balance between personal data protection and data usage for public health interests. Future research directions and implications of the present research are also discussed. 

## 2. The Development of Digital Health, the GDPR, and Personal Health Data Protection

### 2.1. Digital Health and the Challenge of Personal Health Data Protection

Digital health is broadly regarded as healthcare practices using information and communication technologies [13]. It covers a range of electronic or digital process in diagnosis, monitoring, and treatment of people’s health [13]. Hospitals and healthcare institutions have built various kinds of Internet healthcare platforms to provide health content. After registering and logging into these platforms, patients can be allowed to make a diagnosis of their symptoms, search for health management knowledge, and create and store individual electronic health records [11]. In this process, the user-generated content (e.g., user profiles, email account information, click streams, search queries, or personal information from cookies, etc.) can help hospitals and healthcare institutions reach out to potential clients for marketing their medicine products and services [11]. Furthermore, these generated personal health data can be used for medicine research and prediction of people’s health. Prediction of health includes early detection and prevention of diseases, inference of health conditions, identification of risks of illness, and much more [14,15]. Moreover, some hospitals and healthcare institutions have also engaged in the development of smart and portable devices that are applied on the individual’s skin to measure blood pressure, heart rate, sleep quality, pulse and breathing, etc. [15,16,17]. Through such devices, individuals can receive continuous monitoring, aiding in the delivery of personalized medicine [18]. 

However, the sharing of personal health data among health service providers, health professionals, health information networks, and patients, is accompanied by the issue of sensitivity of personal data, which has raised specific concern from personal data protection laws [19]. Out of the respect for the privacy of the patient and the protection of confidentiality in health services, the European Court affirmed that “domestic laws must afford appropriate safeguards to prevent any such communication and disclosure of personal health data” [20]. The European Union data protection regime has set up a special set of principles and rights of health data protection. The European Union Data Protection Directive (DPD) makes explicit reference to the principles of basic health data protection. These principles require that the collection and processing of personal health data must follow the four standards, that is, purpose limitation, data minimization, proportionality, and control. Additionally, the subjects (i.e., patients) are given rights to give consent, access data, be informed of the purpose and logic involved in data processing, rectify the information about them, or object to the data processing in some cases. With the growing capacities of information retrieval in “cloud” servers, and the increasing easiness of sharing and diffusing sensitive personal data on the Internet [21], however, the applicable limitations of the DPD have emerged. It does not apply to the special features of the Internet or the emerging technologies, such as cloud computing and big data very well, although some extensive interpretation on it has been done, and some efforts on the part of the European Court of Justice have been made [11]. This problem drives the emergence of the GDPR, which is viewed as a huge step forward for personal health data protection in the European Union, in order to face future challenges from information technology development [22]. 

### 2.2. Digital Health and Personal Health Data Protection of the GDPR

The GDPR appears much more adequate to respond to challenges of personal health data protection in the digital health era [15]. It provides patients with stronger rights and more power in health data control and ownership [8,9,10], and endeavors to clarify rights and protection of personal health data in digital healthcare exchanges [6,7]. 

The GDPR, at first, gives the more detailed definition of “data concerning health” than before, referring to personal data related to the physical or mental health of a natural person, including all data about his or her past, current, or future health status collected in the course of the registration for or the provision of healthcare services to him or her [15]. More importantly, it establishes new data protection standards relating to health and toughens the obligation of data controllers and processors in the health field. 

To be specific, the GDPR sets the higher standards concerning informed consent and notification duties. As Article 7 states, a patient needs to be informed of the possible risks of data collection “in an intelligible and easily accessible form, using clear and plain language”. In addition, since excessive data collection can make consent invalid, the data controller, before data collection, must state whether the provision of personal health data is required by a law or contract, or whether it is a necessary condition to entry into a contract, whether the patient has an obligation to provide personal data, as well as the potential consequences when such data will not be provided (Article 13). Also, to improve the inefficiency of long and complicated privacy notices, the GDPR advocates for using short text with standardized icons to aid immediate awareness of the intended data processing (Article 12, 13, 14). 

The GDPR also strengthens the protection for the right of access to personal health data. Healthcare organizations are required to respond to patient access requests in a shorter deadline (30 days) than before (40 days). To enhance transparency between patients and healthcare service providers, the GDPR introduces the duty of data protection impact assessment (DPIA). Data controllers are required to carry out a DPIA before processing the sensitive information concerning health to identify the possible risks of data processing and find ways to solve them. Further, some provisions (Articles 12, 13, 22, 29) aim to clarify for patients the decisions made by automated processing, including profiling, and the logic involved in data processing, as well as the consequences. In this way, the risks incurred when decision-making algorithms are used are made transparent to patients, enabling them to exercise human intervention in the decision [8]. The GDPR also introduces cybersecurity provisions to improve transparency. Article 5 gives the general principle of integrity and confidentiality. In the case of data breaches, Articles 33 and 34 require data controllers to inform the supervisory authority within 72 h, and if the data breaches become highly risky, they have to inform the patients. 

Considering the great technical development in the healthcare field can directly cause the increasing loss of “legibility” of health data processing [15], the GDPR makes patients the “joint-controller” of their own health data by empowering them to become the protagonists of their personal health data processing [15,23]. It offers patients a new right of data portability, that is, they are empowered to transfer data provided to a data controller previously to another data controller without hindrance from the previous one (Article 20). The right to ask data controllers and processors to erase their health data is also extended by the GDPR. The patients can make such requests not only to search pages but also in other cases, like social network pages (Article 18). 

In addition, especially for health data collected via wearable devices, privacy by design is viewed as the most important provisions of health data protection in the GDPR [24]. It asks healthcare organizations to apply health data protection from the start (Article 25). Besides, as stated by privacy by default, the highest privacy setting, by default, should be automatically applied to a new service product of health [7]. In case of non-compliance with the GDPR, healthcare organizations will face the higher sanctions. The administrative fines have been massively increased up to 2% of their global annual revenue or €10 million in case of minor breaches, or as high as 4% of their global annual revenue or €20 million in the case of major breaches (Article 83). 

Therefore, with the new standards ranging from informed consent, notification duties, data access, data protection impact assessment (DPIA), algorithmic transparency, automated decision-making, data portability to privacy by design, privacy by default, and high sanctions applying across the European Union, the GDPR increases the level of legal protection for personal health data, making the balance between health data uses (business interests) and personal health data protection (individual interests) lean to the latter [25]. 

### 2.3. The Impact of GDPR Shock on Digital Public Health Dectors across the European Union 

The existing solutions to meet requirements of previous regulations on data protection have not adapted to the new and more stringent standards in the GDPR. Hospitals and healthcare institutions across the European Union thus need to make further adjustments from staff recruitment, staff training, business adjusting, and technical upgrades, etc., by doing so, aim to look for the balance between their profit maximization and effective protection for personal health data. 

The health data protection training for staff needs a further update to cope with this new legislation. In general, most data breaches are internal rather than external hacks; the internal training of data protection is thus necessary for all staff [7]. Inadequate training may result in internal data breaches, which can in turn incur high sanctions from the GDPR and big financial losses to healthcare organizations. 

It is also necessary to recruit staffs that are well qualified for the key roles of the GDPR, such as data protection officers that take charge of informing, advising, and monitoring compliance, data privacy teams, and IT project and program managers. The shortage of such staff is expected to be disruptive to healthcare organizations with the digital health as the primary business [7]. They should take action sooner to prevent more serious staff shortage incurred by the increasingly fierce competition for staff resources near the time of the GDPR’s implementation. 

Moreover, healthcare organizations should review how their current data have been collected as soon as possible. Early evaluation can guide them in how to address the challenges of the GDPR. For those that find themselves unable to deal with the GDPR, it may be the better practice to leave the current market to avoid serious financial losses, and then create business in accordance with new survival opportunities. 

Information technologies used in personal health data protection are also needed to display advanced functions that can meet the new GDPR requirements, such as carrying out data protection impact assessment (DPIA), new specifications for consent and notification, new deadlines for data breaches (72 h), reduced processing time for data access requests (30 days), and the other measures mentioned earlier.

These devoted efforts in improving personal health data protection can bring more business opportunities for healthcare organizations in the long term, however, huge costs are incurred to fulfil all these adjustments [7]. Hence, the policy shock of the GDPR to financial performance of healthcare organizations will be inevitable in the short term. 

### 2.4. The Evaluation on the GDPR’s Effectiveness for Health Data Protection

This data protection reform is, for some, far beyond the scope of any previous experience. The effectiveness of this legal policy for health data protection can be examined through testing whether the pre–post change in hospitals’ financial performance is significantly negative. This is based on the fact that the GDPR proposes higher requirements of health data protection, which in turn inevitably induces greater input for hospitals and thus causes a shock to their financial performance.

In this study, considering the digital health business is not viewed equally important for all hospitals across the EU, financial performance seems to be highly vulnerable to the policy shock of the GDPR in those institutions that consider digital health service as the largest primary business except for traditional hospital services, whereas those without or with the digital health service as only a small proportion of their operating business are is less likely to suffer from this shock. Accordingly, comparing the pre–post change in financial performance between these two groups can test the policy effect of the GDPR on promoting health data protection in the digital health age.

## 3. Method

### 3.1. Sample and Measures

Our sample comes from Bureau Van Dijk (BVD)-Amadeus. The Bureau Van Dijk is a part of the database of Moody’s Analytics, a top-3 rating institution located in the United States. This database reports on the financial performance of hospitals in EU-28 countries using the NACE classification. The NACE classification is the industry standard classification system used in the European Union, and is the abbreviation of the French *Statistique des activités économiques dans la communauté européenne* (Statistical Classification of Economic Activities in the European Community). The database can be accessed via the website https://amadeus.bvdinfo.com/. The sample period of this study is 2013–2017. The return of asset (ROA, based on operating revenue scaled by total assets) is used to measure hospitals’ financial performance [26]. In line with previous research, this study controls leverage, firm size, cash flow, and cash holdings that serve as important determinants of hospitals’ financial performance [27,28]. More specifically, leverage is controlled since higher leverage reflects the presence of closer relationships between hospitals and financial intermediaries (e.g., commercial banks, insurance companies, pension funds), which enables hospitals to acquire a market information advantage and then facilitates their financial performance [27]. Firm size is controlled considering it is related to economy of scale and thus may positively affect hospitals’ financial performance [29]. Cash flow is controlled considering that lower cash flow suggests that hospitals are faced with deteriorated operation status (e.g., the lower occupation rate, and slower collection of receivables) and thus may affect hospitals’ financial performance [28]. Cash holding is controlled considering that it is important for sustaining sound hospital operation and thus may affect hospitals’ financial performance [30].

### 3.2. Research Design 

The difference-in-difference (DID) approach is widely used as the effective way to investigate the policy influence. The core idea of DID approach is to test the policy influence on outcomes by evaluating the significance of pre–post change (change in outcomes before and after the policy). In the DID design, two differences are important: (1) the difference of the outcome variable after vs. before the policy change in the group exposed to the policy (named B2-B1), and (2) the difference of the outcome variable after vs. before the date of policy change in the group unexposed to the policy (named A2-A1). The DID approach is to separate out the policy influence by evaluating the outcome change related to the policy implementation beyond the background change, that is (B2-B1) – (A2-A1). 

Statistically, the policy influence on outcomes is tested through the interaction term between the pre-post and exposed-unexposed variables. The policy influence is statistically present if the estimated parameter of interaction term is significantly different from zero.

According to the DID approach, in this study, hospitals with the information, communication, and telecommunication business surrounding health as their largest business except for the provision of common hospital services are viewed as the treatment group (NACE Rev.2 secondary 61, 62, 63), and the rest of the hospitals are correspondingly viewed as the control group. Thus the control group not only includes hospitals without the digital service, but also those that do not take it as the secondary business just behind the common hospital service. This study uses the DID approach to compare the financial performance difference between these two groups. The period 2013–2015 is defined as the pre-legislation period and 2016–2017 is defined as the post-legislation period. Thus, there are 24,637 valid hospital-year observations in the control group and 1289 valid hospital-year observations in the treatment group after excluding missing values in dependent and control variables (more details are shown in Table A1). Specifically, our regression model is as follows.

(*financial performance*)*_it_* = *β*_1_ + *β*_2_(*treatment group*)*_it_* + *β*_3_(*post legislation*)*_it_* + *β*_4_(*treatment group* × *post legislation*)*_it_* + *β*_5_(*control variables*)*_it_* + *ε_it_*

In the above regression equation, the subscript *i* indicates the *i*-th hospital, and the subscript *j* indicates the *j*-th year. The two-dimensional panel regression contains both cross-sectional and time series information. The variable “treatment group” is a 0–1 binary variable. The binary variable “treatment group” = 1 if the hospital belongs to the treatment group, and = 0 if the hospital belongs to the control group. The variable “post legislation” is also a 0–1 binary variable. The binary variable “post legislation” = 1 if the year is 2016 or after, and = 0 if the year is 2015 or before. As such, the interaction term “treatment group × post legislation” equals 1 only when the sample point indicates that the hospital belongs to the treatment group and the time is 2016 or after. Our main interest is the coefficient of the interaction term (*β_4_*) which captures the change in financial performance of the treatment hospitals relative to the control hospitals subsequent to the legislation of the GDPR. A significant negative (positive) coefficient of *β_4_* indicates a decrease (increase) in financial performance after the legislation.

### 3.3. Potential Self-Selection Bias Issue and the Use of Propensity Score Matching (PSM)-DID Procedure

To clarify the concern of potential self-selection bias, this study re-examines the above treatment effect using the combination of the propensity score matching (PSM) and difference-in-difference (DID) procedure. The PSM-DID procedure has been widely used to examine the causal treatment effect and is appropriate to deal with the potential self-selection bias problem [31,32,33,34].

Under the background of this study, the potential self-selection bias represents that the hospitals with poorer financial performance at the beginning may be more motivated to provide digital services to attract patients compared to their counterparts, and thus their poorer financial performance is pre-determined and preexisting, regardless of whether the legislation of the GDPR is in force or not. According to previous studies, the decision of a public health institution to provide digital health services depends on its capital input, human input, cash flow and leverage [35,36,37]. More specifically, hospitals with more capital and human input have more financial resources to allocate and thus the likelihood of their investing in the provision of digital health services increases [37]. Higher cash flow implies a larger amount of patients’ visits to hospitals, and in this case, hospitals are more motivated to provide digital services to lower their operational costs [35]. Higher leverage reflects higher debt level of hospitals, and the shortage of financial resources can make hospitals hesitate to invest in digital service provision [35,38].

Following the PSM-DID procedure, this study uses these variables as matching variables to predict the propensity score and re-match the new control and treatment group. The matching variables should not display significant differences between the new control and treatment group. The newly matched control and treatment group will meet the requirement that whether a hospital decides to provide digital services or not is quasi-random. After that, the use of DID on the new matched groups no longer suffers from any possible self-selection bias theoretically. 

More specifically, the above procedure can be clearly divided into three steps. In Step 1, the logit regression is used to obtain the estimated propensity scores, where the binary dependent variable is whether or not the hospital takes the digital health as the largest business except for the common hospital service. The estimated propensity scores enable us to construct a new matched treatment group and a control group that are similar in the matching variables. The main matching method used in this study is “nearest-neighbor (NN) matching”, which takes the observation with the closest propensity score for comparison. The reason why the nearest neighbor is chosen rather than other matching methods is that it allows the repetitive use of objects for matching, and thus is capable to overcome the shortcoming of inefficiency and matching bias induced by one-to-one matching [31]. Such a feature is important for reducing matching bias when there are several observations with very close propensity scores and all scores are within the matching tolerance limit. For one-to-one matching, only the most closest can be matched and others are left unmatched. As suggested by previous studies, nearest-neighbor matching with replacement is better than that without replacement [31]. In the former case, an untreated individual can be used more than once as a match, whereas in the latter case it is considered only once. The average quality of matching will increase and the bias will decrease if the replacement is allowed in matching [31]. In this study, we allow the replacement to be three and seven, respectively, which means that each untreated individual can be used three times and seven times respectively in the matching. In Step 2, this study verifies that the propensity scores for the new matched treatment and control group lie in an overlap of common support, and this study conducts the balance check to detect whether there is significant difference between the two groups after matching. In Step 3, the common DID approach is used to estimate the difference in financial performance between these two matched groups throughout the sample period. In this way, the treatment effect of the policy on financial performance is examined.

### 3.4. Placebo Tests

Further, this study performs a series of placebo tests. The purpose of these tests is to examine the sensitivity of DID results [39,40,41]. Specifically, this study needs to assume the pseudo legislation years, 2015 and 2014, and re-examines the treatment effect. The estimated coefficient (*β_4_*) of the interaction term “treatment group × post legislation” is expected to not be significant when assuming a pseudo legislation year. Besides, this study also re-examines the treatment effect when deleting the first legislation year 2016 and retaining the post legislation year 2017. The estimate of the interaction term is expected to remain significant if the treatment effect is robust.

## 4. Empirical Results

### 4.1. Main Results

Table 1 reports the results of the standard difference-in-difference (DID) regression, and shows that the legislation of the GDPR imposes negative impact on financial performance of hospitals with the digital health service in the European Union (−0.3589, −1.0630, *p* < 0.05, Panel A). The propensity score matching difference-in-difference (PSM-DID) result shows that the above influence remains robust and significantly negative (−0.1651, −0.1616, *p* < 0.01, Panel A). Besides identifying the existence of the treatment effect, we also provide an estimate of the magnitude of the effect (the difference = −0.6335, −0.5959, *t*-statistics = −2.1900, −2.4000, *p* < 0.01, Panel B, for the average treatment effect for the treated (ATT)). For the PSM-DID regression, the results of matching quality check of variables are also presented (see Table A2 in Appendix A). Results of matching quality check show that there are no significant differences between the treatment and control group in matching variables (i.e., capital input, human input, cash flow, leverage) that may affect hospitals’ intention to provide the digital service and take it as the second major business besides common hospital activities. These results demonstrate that the above PSM-DID result is reliable.

### 4.2. Placebo Test Results

Because of the complexity of real social environment, it is almost impossible to design a test under a completely controllable laboratory environment to validate the causal link between the policy and the change of outcomes [42,43]. As such, the “placebo test” to check policy sensitivity is often used in the field of health economics and policy evaluation. The reason why the robustness check for policy sensitivity is called the “placebo test” is that it is in line with the core idea of the “quasi experiment”. 

More specifically, if the outcome change is indeed incurred by the policy implementation, the estimated parameter of interaction term “treatment group × post legislation” should not be significant when setting the pseudo legislation year before the real one. In the background of this study, the failure to find the significant effect of interaction term on the outcome variable when assuming the pseudo-legislation year is 2015 suggests that there is no other factors influencing the outcome variable and the outcome change is uniquely affected by the policy implementation. Besides, when deleting the first legislation year 2016 and leaving others unchanged, the interaction term should remain significant if the policy influence indeed exists and continues after the legislation year.

Results of placebo tests are shown in Table 2. The influence of the legislation of the GDPR on hospitals’ financial performance recedes into insignificance regardless of whether the pseudo legislation year is 2015 (−0.4589, *p* >0.05, number of hospital-year observations = 25,926) or 2014 (−0.6586, *p* >0.05, number of hospital-year observations = 25,926). In the last column, this study repeats previous analysis when deleting the first legislation year 2016, and finds that the interaction term “treatment group × post legislation” remains significantly negative (−0.3593, *p* < 0.05, number of hospital-year observations = 20,498). These results confirm the treatment effect of GDPR policy is robust and insensitive. 

## 5. Discussion, Implication, and Conclusions

### 5.1. Discussion

The empirical results validate the effectiveness of the GDPR for health data protection. By identifying the negative pre–post change of hospitals’ financial performance, this study tests and confirms the effectiveness of the policy. Based on the fact that much greater financial input than ever before is needed to meet the stricter requirements of data protection, a subsequent decline of financial performance will inevitably happen to hospitals that attach great importance to digital health services.

Since hospitals do not equally emphasize the digital health business, the GDPR that urges greater financial input for stricter health data protection is more likely to impact the financial performance of hospitals that view the digital health service as the largest primary business except for traditional hospital services. In contrast, for hospitals without or just having the digital health service as a small share in their operating business, the influence of the GDPR will be much smaller. Through identifying the negative pre–post change of financial performance of hospitals in the treatment group compared with those in the control group, the effectiveness of the GDPR for health data protection can be validated.

### 5.2. Managerial and Academic Implications

This study has several managerial and academic implications. First, the GDPR by its very nature has captured much attention from industry and academics alike. Due to its relative modernity and novelty, there is no vast body of academic literature present, although articles and opinion pieces discussing it are fairly numerous. So far, there are no deeply empirical studies into organizational compliance with this new regulation. Most relevant studies appear to focus on reporting the concrete preparations made for compliance, for example, the budget sizes expended or the increase in workforce size. There is yet to be in-depth empirical research to precisely test whether this new regulation is effectively implemented (or complied with) by organizations, and what the actual extent of the compliance has been. This study addresses that gap. 

Second, in recent years, digital health has become a forefront issue in the health field. Much attention has been devoted to the important role of digital health services in attracting potential and retaining existing patients. For example, digital health services have been found as a good channel to improve patients’ adherence to a range of healthcare practices, including interaction with health service providers, engagement in medical consultations, online health information seeking, and self-monitoring of health [44,45]. With the recurring health data breaches resulting from internal sharing, data mining, precise marketing, and data exchange, etc. [46], however, potential data security hazards of digital health have become increasingly obvious. As such, the issue of health data protection has started to attract increasing attention from healthcare organizations, researchers, and legislators. To enrich the relevant research, this study empirically examines the influence of the newly legislated GDPR on the financial performance of fast growing digital public health sectors. In this way, this study can help understand the actual extent of their attempts in protection for patients’ health data. 

Third, this study is the first attempt to empirically examine the policy effect of the GDPR, the latest legislation on data protection, on digital public health sectors across the European Union. The timely evaluation of this policy influence can give legislators and healthcare organizations the timely and valid findings, helping them promote the GDPR’s implementation. The decline of financial performance suggests that digital public health sectors are making costly adjustments to the GDPR’s requirements of patients’ personal health data protection, which demonstrates the implementation of this new regulation have achieved preliminary success. However, this finding also reflects the existing conditions of design and deployment of healthcare technologies in these organizations have not fully met the new standards of personal health data protection required by this new regulation. Thus, European Union legislators need to further promote the level of GDPR implementation, and digital public health sectors across the European Union need to make urgent and further adjustments in the design and deployment of healthcare technologies to ensure the balance between personal data protection and data usage.

### 5.3. Limitations and Future Research

This study is not free of limitations. First, due to the availability of data, this study still could not answer how long this negative impact lasts. The issue can help understand the whole course of the GDPR’s implementation by digital public health sectors across the European Union. Thus, as new data become available, future research can investigate this issue to complement our analysis. 

Second, given the huge cost expended in adjustments to the GDPR, it is necessary to investigate whether or not this policy shock would deter the new entry of hospitals into the digital public health market. Future research can use new data to make deeper explorations in this field. 

Third, this new regulation is designed to protect the health privacy of European Union citizens, regardless of the location of their health data, and it gives European Union courts the power to severely punish any healthcare organizations in the world that misuse European Union citizens’ health data. As such, the sample of this study focusing on the public health sectors across the European Union may limit the application of research findings at the local level. Future research can make extensions by using global samples to investigate the policy effect of the GDPR. 

### 5.4. Conclusions

Digital health has become an increasingly important trend and has been highly emphasized by medical and healthcare institutions. The policy effect of the General Data Protection Regulation (GDPR) was found to be effective in preventing a savage development of this wave of digitalization. The digital public health sectors across the European Union have made some costly adjustments to comply with this new regulation that requires much more stringent protection for patients’ health data. Their financial distress may reflect the huge cost of re-adaptation to this new regulation. Meanwhile, this financial distress also reveals the fact that the protection for patients’ personal health data has been insufficient in the past. If digital public health sectors already have enough input and support in the protection for patients’ health data before the unexpected legislation, this new legislation on data protection may not cause a sudden decline in their financial performance. Moreover, the negative impact of the GDPR on financial performance could be the watershed of digital public health development. It forecasts the beginning of a shuffle of digital public health markets, and only those institutions capable of affording the capital and human input in the effective protection for patients’ personal health data will survive in the future.

## Figures and Tables

**Table 1:** The influence of the General Data Protection Regulation (GDPR) on financial performance of hospitals in the European Union.

	Panel A. Regression Analysis-Dependent Variables: Financial Performance				
	Standard DID	Standard DID	PSM-DID (*nearest-neighbor = 3*)		PSM-DID (*nearest-neighbor = 7*)
Independent variables					
Treatment group × Post legislation	−1.0630 * (0.3675)	−0.3589 * (0.1814)	−0.1651 ** (0.0472)		−0.1616 ** (0.0450)
Control variables					
Post legislation	0.6984 (0.4210)	−0.0134 (0.0647)	−0.0426 ** (0.0106)		−0.0413 ** (0.0101)
Treatment group	−1.0752 ** (0.3675)	−0.1713 (0.1654)	N.A.		N.A.
Hospital size		−0.0066 ** (0.0020)	−0.0482 ** (0.0060)		−0.0610 ** (0.0070)
Leverage		−0.0001 ** (0.0000)	−0.0000 (0.0000)		−0.0000 (0.0000)
Cash holding		−0.0708 ** (0.0123)	0.0123 (0.0235)		0.0009 (0.0238)
Cash flow		0.0162 (0.0130)	0.0466 ^†^ (0.0266)		0.0649 ^†^ (0.0316)
Intercept	2.4925 ** (0.3417)	1.6008 ** (0.0284)	1.6145 ** (0.0122)		1.6265 ** (0.0131)
Hospital fixed-effect	No	No	Yes		Yes
Sample size (hospital-year)	34,291	25,926	23,606		22,697
F-statistics (*p*-Value)	30.77 (0.0000)	25.73 (0.0000)	30.04 (0.0000)		34.87 (0.0000)
	**Panel B. PSM (Average treatment effect for the treated estimate)**				
	Treated	Control	Difference	S.E.	*t*-statistics
ATT (Nearest neighbor3)	1.6495	2.2830	−0.6335	0.2886	−2.1900 **
ATT (Nearest neighbor7)	1.6494	2.2454	−0.5959	0.2479	−2.4000 **

Notes. ATT: average treatment effect for the treated. Financial performance is computed by operating revenue scaled by total assets. Hospital size is measured by total assets (in €10 million). Leverage is computed as total assets/shareholder funds. Cash holding and cash flow are both in €10 million. Robust standard errors are reported in brackets. Off-support observations are not included in the PSM-DID regression. The estimated parameter of treatment group in the third and fourth column is automatically omitted by the software as the number of hospitals belonging to the new matched treatment group is not large enough and quasi-collinear with other terms. ^†^ *p* < 0.10, * *p* < 0.05, ** *p* < 0.01. DID: difference-in-difference; PSM: propensity score matching.

**Table 2:** Placebo tests.

	Dependent Variables: Financial Performance		
	Pseudo Legislation Year Being 2015	Pseudo Legislation Year Being 2014	Deleting the First Legislation Year 2016
Independent variables			
Treatment group × Post legislation	−0.4589 (0.2510)	−0.6586 (0.4761)	−0.3593 * (0.1783)
Control variables			
Post legislation	0.0378 (0.0495)	0.0441 (0.0422)	−0.0784 (0.0399)
Treatment group	−0.0399 (0.2438)	0.2107 (0.4735)	−0.1727 (0.1654)
Hospital size	−0.0066 ** (0.0020)	−0.0066 ** (0.0020)	−0.0076 ** (0.0023)
Leverage	−0.0001 ** (0.0000)	−0.0001 ** (0.0000)	−0.0001 ** (0.0000)
Cash holding	−0.0712 ** (0.0124)	−0.0712 ** (0.0123)	−0.0662 ** (0.0145)
Cash flow	0.0163 (0.0131)	0.0162 (0.0130)	0.0174 (0.0134)
Intercept	1.5743 ** (0.0214)	1.5611 ** (0.0256)	1.6023 ** (0.0289)
Number of hospital-year observations	25,926	25,926	20,498
F statistics (*p*-Value)	28.26 (0.0000)	25.62 (0.0000)	21.10 (0.0000)

*Notes:* Standard difference-in-difference is implemented. Robust standard errors are reported in brackets. * *p* < 0.05, ** *p* < 0.01.

## Abbreviations

EU	European Union
GDPR	general data protection regulation
ICT	information and communication technology
BVD	Bureau Van Dijk
NACE	*Statistique des activités économiques dans la communauté européenne* (Statistical Classification of Economic Activities in the European Community)
DID	difference-in-difference
PSM	propensity score matching

## Appendix A

**Table A1:** An overview of variables (original sample).

	Treatment Group			Control Group		
	*N* (Hospital-Years)	Mean	S.D.	*N* (Hospital-Years)	Mean	S.D.
Financial performance	1289	1.2763	3.5992	24637	1.5594	4.2843
Hospital size	1289	0.3953	2.0217	24637	3.0549	14.6101
Leverage	1289	4.0077	22.939	24637	5.3876	196.477
Cash holding	1289	0.0405	0.1791	24637	0.2611	1.1684
Cash flow	1289	0.0178	0.0975	24637	0.1618	1.4109

Notes: Financial performance is computed by operating revenue scaled by total assets. Hospital size is measured by total assets (in €10 million). Leverage is computed as total assets/shareholder funds. Cash holding and cash flow are both in €10 million. Observations with missing values of above variables are omitted. The sample period is 2013–2017. The data from BVD-Amadeus were updated in December 2018.

**Table A2:** The matching quality of propensity score matching (PSM).

	Panel A. Propensity Score Prediction-Dependent Variables: (=1 if the Hospital Taking Digital Business as the Largest Business Except for Common Hospital Services, Otherwise = 0)				
	Parameter			Standard Error	
Matching variables					
Capital input	−0.4492 **			(0.0681)	
Human input	0.0974			(0.0830)	
Cash flow	−0.8048 **			(0.2186)	
Leverage	0.0001			(0.0002)	
Intercept	−2.8063 **			(0.0495)	
LR χ2 (*p*-Value)	229.66 (0.0000)				
Matching period (before legislation)	2013–2015				
Observation size	15,098				
	**Panel B. Balance check [nearest-neighbor, *N* = 3]**				
	Mean			*t*-test	
	Treated	Control	Bias %	T-value	*p* > *t*
Capital input	0.5117	0.4839	0.3	0.22	0.8260
Human input	0.3126	0.2710	1.2	0.52	0.6020
Cash flow	0.0222	0.0328	−0.9	−1.31	0.1900
Leverage	4.8218	−3.4042	5.0	0.80	0.4230
	**Panel C. Balance check [nearest-neighbor, *N* = 7]**				
	Mean			*t*-test	
	Treated	Control	Bias %	T-value	*p* > *t*
Capital input	0.5117	0.4839	0.3	0.22	0.8260
Human input	0.3126	0.2540	1.6	0.77	0.4420
Cash flow	0.0222	0.0344	2.5	−1.07	0.2860
Leverage	4.8218	0.7040	−0.7	0.60	0.5460

**Notes:** Logistic model is applied for the propensity score prediction. Standard errors are reported in brackets. Capital input is measured by total assets, and human input is measured by total costs of employees of hospitals (both in €10 million), * *p* < 0.05, ** *p* < 0.01.
